# Within-country migration and obesity dynamics: analysis of 94,783 women from the Peruvian demographic and health surveys

**DOI:** 10.1186/s12889-019-6586-7

**Published:** 2019-03-04

**Authors:** Hector Najera, Shailen Nandy, Rodrigo M. Carrillo-Larco, J. Jaime Miranda

**Affiliations:** 1 University of Bristol, Bristol, UK; 2 Cardiff University, Cardiff, UK; 3 CRONICAS Centre of Excellence in Chronic Diseases, Universidad Peruana Cayetano Heredia, Lima, Peru; 4 School of Medicine “Alberto Hurtado”, Universidad Peruana Cayetano Heredia, Lima, Peru

**Keywords:** Migration, Obesity, Urbanization, Demographic and health surveys

## Abstract

**Background:**

Rural-to-urban migration is associated with increased obesity, yet it remains unknown whether this association exist, and to what extent, with other types of internal migration.

**Methods:**

We conducted a secondary analysis of the Peruvian Demographic and Health Surveys (2005 to 2012) on data collected from women aged 15–49 years. Participants were classified as rural stayers, urban stayers, rural-to-urban migrants, intra-rural migrants, intra-urban migrants, and urban-to-rural migrants. Marginal effects from a logit regression model were used to assess the probabilities of being and becoming obese given both the length of time in current place of residence and women’s migration status.

**Results:**

Analysis of cross-sectional survey data generated between 2005 and 2012. Data from 94,783 participants was analyzed. Intra-urban migrants and rural-to-urban migrants had the highest rates of obesity (21% in 2012). A steady increase in obesity is observed across all migration statuses. Relative to rural non-migrants, participants exposed to urban environments had greater odds, two- to three-fold higher, of obesity. The intra-rural migrant group also shows higher odds relative to rural stayers (42% higher obesity odds). The length of exposure to urban settings shows a steady effect over time.

**Conclusion:**

Both exposure to urban environments and migration are associated with higher odds of obesity. Expanding the characterization of within-country migration dynamics provides a better insight into the relationship between duration of exposure to urban settings and obesity.

## Background

Over the last 40 years, there has been a significant increase in obesity, reflected by a high body mass index (BMI); between 1975 and 2014, global prevalence increased by 7.6 and 8.5% in men and women, respectively [1]. This pattern of increase however, is not uniform across countries or within countries, with different groups at greater or lesser risk both of becoming obese and also succumbing to associated health problems. A recent paper from Peru, using panel survey data on rural-to-urban migrants collected between 2007-08 and 2012–13 found that, compared to the rural group, rural-to-urban migrants and urban dwellers had an almost ten-fold greater risk of developing obesity [2]. Such discrepancies could be due to socio-economic differences, which determine exposure to known obesogenic risk factors such as sedentarism (low level of physical activity), limited access to healthy food choices among the worse-off [3], and stress [4, 5].

Although the risk factors for, and health consequences of, obesity are well known, how the probabilities of being obese vary according to different within-country internal migration profiles, and how these relationships behave over time, remains unknown in most low and middle-income countries (LMICs). Rapid urbanization is placing an undeniable strain on public resources in LMICs, and urban environments provide a range of challenges to newcomers from rural settings, particularly with regards to health and nutrition [6].

Collecting reliable longitudinal data in LMICs is expensive and difficult, and remains a challenge, particularly in settings where rural-to-urban migration is widespread, rapid and regular. Using repeated cross-sectional population-based surveys could be a pragmatic option to gain information both on migration profiles and health indicators. The aim of this paper is to assess the probabilities of being obese for different profiles of internal migration in Peru using the Peruvian Demographic and Health Surveys (DHS) from 2005 to 2012 [7]. In doing so we show how the use of cross-sectional household survey data can reflect patterns of obesity, and its relationship with migration, and could be used in many more LMICs.

## Methods

### Study design

Secondary-data analysis using repeated cross-section surveys from the Peruvian DHS conducted annually between 2005 and 2012. All datasets for the years 2005 to 2012 were pooled to increase power. The exposure variable (migration status, see below) is a nominal variable with small sample sizes for some categories and, therefore, a single year lacked sampling power to make a sensible inference.

### Study population

Data on obesity and migration as well as residence status were obtained from women aged 15–49 years (Table 1). Excluded from analyses were visitors to the household and also the very small number of migrants from overseas who reported moving to rural areas.

**Table 1:** Summary statistics. Peru. 2005–2012, women aged 15–49

	2005	2007	2008	2009	2011	2012
Sample size	5803	5932	14,957	22,969	21,761	23,361
Obesity (Prevalence %)	13	15	14	16	17	18
Age (Mean years)	30	31	31	31	31	31
Education (Mean years of schooling)	9	9	9	10	10	10
Wealth Index (Mean quintile)	3	4	4	3	3	3
Duration in place of residence	22	22	22	22	21	22
(Mean years)						
Migration status	% (Column)					
Urban stayers	41	39	42	43	40	41
Intra-urban migrants	22	23	23	24	25	24
Rural stayers	20	18	17	15	14	13
Rural-to-Urban migrants	6	10	9	8	10	10
Intra-rural migrants	5	6	5	5	6	6
Urban-to-Rural migrants	7	5	5	5	5	6
Ethnicity (language)	% (Column)					
Spanish speaker	96	95	96	96	96	95
Quechua	2	5	4	3	4	4
Aymara	1	0	0	1	0	1
Other	0	0	0	0	0	0

Standard deviations are almost constant across years for the continuous variables:

Age (9.9–10.1); Education (3.8–4.3); Wealth index (1.3–1.4); Duration in place of residence (12.5–12.8)

### Variables

#### Primary exposure variable

A derived variable to reflect migration status of respondents was created using information on their current place of residence, their childhood or previous place of residence, their current age, and the number of years lived in their current place of residence. This information was used to create six categories of resident status: (i) rural stayers (i.e., resident in the same rural area for all of their lives), (ii) urban stayers (i.e., resident in the same urban area for all of their lives), (iii) rural-to-urban migrants, (iv) intra-rural migrants (i.e., respondents who reported moving from one rural area to another), (v) intra-urban migrants (i.e., respondents who reported moving from one urban area to another), and (vi) urban-to-rural migrants (i.e., respondents who reported moving from an urban to a rural area).

#### Outcome variable

The outcome of interest was body mass index (BMI), calculated from height and weight measurements (Kg/m^2^). Respondents with a BMI of 30 Kg/m^2^ or more were classified as obese [8].

#### Auxiliary variables

Auxiliary variables included household wealth index, based on information on ownership of assorted assets and access to services, such as water source and form of sanitation [9]; level of education (years of education); ethnicity (language spoken at home), and length of time in current place of residence (in years). The participant’s age is presented in years. We also controlled for geographical region and altitude of respondent’s current place of residence [10]. These two variables were used as potential confounders in the analysis.

### Statistical analysis

For descriptive purposes, numeric variables were summarized with means, and categorical variable were presented as percentages along with 95% Confident Intervals (95% CI). A multivariable logit model was utilized to analyze the relationship between obesity and the exposure variable. Marginal effects (Adjusted Predictions at the Means) were computed from the logit model to obtain the predicted probabilities of being obese, given the length of current place of residence, with rural stayers, i.e. respondents who reported living in a rural location for all their lives and never migrating, set as the reference group. Survey weights and year of the survey were utilized to adjust the estimates and capture between-survey differences. The models were fitted using the pooled dataset 2005–2012. To explore whether the migration groups changed in terms of socioeconomic profiles over time, we plotted their mean asset index scores for the years 2005 to 2012 without observing major differences.

## Results

### Sample characteristics

Data from 94,783 women was analyzed and sample size ranged from 5803 to 23,361 participants in 2005 and 2012, respectively (Table 1). Average age of respondents at the time of each survey was 30 years, and the mean duration in their place of residence at the time of the interview was 22 years. There were two apparent changes in the composition of the sample, with the share of rural stayers falling from 20% in 2005 to 13% in 2012; in contrast, the share of rural-to-urban migrants increased from 6 to 10% over the same period. Overall obesity prevalence among women increased from 13% [95% CI 12–14%] in 2005 to 18% [95% CI 17–19%] in 2012. All other variables remained stable over the seven-year period (Table 1).

### Migration and obesity

The prevalence of obesity by migration status (Table 2) shows two clear patterns. First, the prevalence of obesity has substantially increased in all study groups between 2005 and 2012, except for urban-to-rural migrants whose confidence intervals overlap, meaning differences in means are not statistically significant. The overall increases in obesity prevalence ranged between 20% and 46% with respect to the 2005 prevalence estimates, but there was almost a doubling in the prevalence of obesity among intra-rural migrants. On its own this finding would be a concern, but it is mitigated somewhat by the fact that obesity amongst this group is relatively low (13% in 2012). Second, there is a gradient in the prevalence estimates: rural stayers have the lowest rates of obesity, followed by intra-rural migrants, urban-to-rural migrants, urban-stayers, intra-urban migrants, and rural-to-urban migrants; this gradient was observed in all study years.

**Table 2:** Obesity prevalence rate (%, and 95 CI) by migration status

Year	Rural stayers	Intra-rural migrants	Urban to rural migrants	Urban stayers	Intra-urban migrants	Rural to urban migrants
2005	6.7	6.6	11.8	13.7	17.0	15.2
	[5.2–8.3]	[3. 5–9. 8]	[8.45–15.2]	[12.2–15.1]	[14.7–19.1]	[10.9–19.3]
2007	7.6	8.6	10.2	15.0	20.8	21.6
	[6.0–9.2]	[5.6–11.5]	[6.67–13.6]	[13.5–16.3]	[18.6–22.9]	[18.2–24.9]
2008	7.7	11.8	11.6	13.9	19.0	20.4
	[6.6–8.9]	[9.4–14.1]	[9.18–14.0]	[13.0–14.7]	[17.6–20.4]	[18.0–22.6]
2009	8.5	11.3	14.5	16.5	18.6	20.1
	[7.6–9.5]	[9.5–13.0]	[12.4–16.5]	[15.7–17.2]	[17.5–19.6]	[18.2–21.9]
2011	9.3	12.8	14.3	17.5	18.7	25.1
	[8.3–10.4]	[11.0–14.6]	[12.2–16.4]	[16.7–18.3]	[17.6–19.7]	[23.2–26.9]
2012	9.8	13.0	15.3	18.8	20.5	21.0
	[8.7–10.8]	[11.2–14.7]	[13.2–17.2]	[18.0–19.6]	[19.4–21.5]	[19.3–22.7]
2012 vs 2005 increase^a^	46.3%	97%	–	37.2%	20.6%	38.2%

^a^These increases are calculated with respect to 2005 values. Figures not calculated for urban-to-rural migrants because confidence intervals for 2012 and 2005 overlap

The multivariable logit model (Table 3) indicate that, relative to rural stayers, all other groups had higher odds of being obese; these odds were almost three times higher in the two groups migrating to urban areas (i.e. intra-urban, and rural to urban migrants). There is also a positive relationship between years lived in place of residence and the probabilities of being obese. Relative to year 2005, the odds of being obese increased in most recent years, being up to 46% higher in 2012. Belonging to a non-Spanish speaking community (i.e. Quechua and other indigenous language speakers) had a protective effect, with around half the risk of being obese in all language groups except for Aymara speakers.

**Table 3:** Multivariable logit model for obesity

	Obesity	
	Odds ratio	95% CI
Migration status		
Rural stayers	Reference	
Intra-rural migrants	1.42***	**[1.22–1.65]**
Urban to rural migrants	2.22***	**[1.91–2.58]**
Urban stayers	2.54***	**[2.28–2.83]**
Intra-urban migrants	2.90***	**[2.50–3.36]**
Rural to urban migrants	2.84***	**[2.45–3.29]**
Year		
2005	Reference	
2007	1.17*	**[1.01–1.38]**
2008	1.07	[0.95–1.23]
2009	1.27***	**[1.13–1.44]**
2011	1.46***	**[1.25–1.59]**
2012	1.46***	**[1.29–1.63]**
Ethnicity (language)		
Spanish speaker	Reference	
Quechua	0.48***	**[0.43–0.54]**
Aymara	0.82	[0.63–1.06]
Other indigenous	0.26***	**[0.14–0.49]**
Other (foreign)	0.15*	**[0.03–0.80]**
Constant	0.01***	**[0.01–0.01]**
Sociodemographic variables		
Age in years	1.06***	**[1.06–1.07]**
Wealth index	1.09***	**[1.06–1.12]**
Education (in years)	0.95***	**[0.94–0.96]**
Years lived in place of residence	1.01***	**[1.01–1.02]**

* *p* < 0.05, ** *p* < 0.01, *** *p* < 0.001. The model included region and altitude but these were omitted from the output

### Marginal effects

**Fig. 1:**
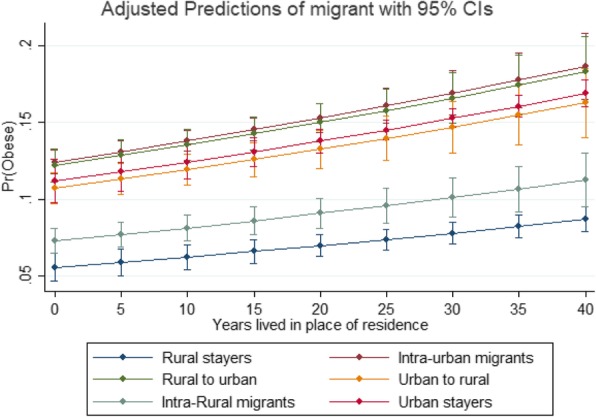
Marginal effects (predicted probabilities of being obese). All migration categories

**Fig. 2:**
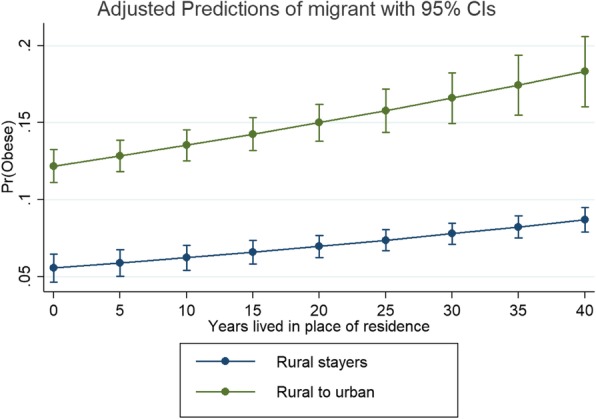
Marginal effects (predicted probabilities of being obese). Rural to urban migrants and rural stayers

Figure 1 and Fig. 2 show the relationship between migration status and the likelihood of being obese, by years lived in place of residence and adjusting by the mean values of the other variables (i.e. age, mean number of years of education, ethno-linguistic group, and region and altitude of residence). Figure 1 shows the predicted probabilities (obtained from the model) of being obese for all internal migration categories according to years lived in the place of residence. The plot suggests that migration to urban sites might have a higher effect on obesity than migration to rural areas. Although the gradient looks similar, the change overtime is significantly different across groups. For example, relative to rural stayers, rural-to-urban migrants are slightly more likely to become obese overtime (1% per year). Figure 2 compares these estimates for rural stayers and rural-to-urban migrants, with the latter showing greater obesity odds regardless of time lived in place of residence. Overall, the plots suggest little differences when accounting the years lived in place of residence. However, as the lines are not parallel, this indicates there is an effect over-time: the slightly steeper *gradient* for urban-to-rural migrants suggests a faster rate of becoming obese, compared to the other groups. This confirms the findings of work done in Peru using panel data [2].

## Discussion

Most studies have characterized BMI levels and obesity rates in national aggregates, stratifying results by rural and urban settings. Yet, the nature of demographic change in most LMICs today means population distributions are changing rapidly as a result of ongoing internal migration processes. In this study we aimed to characterize obesity rates by expanding into a more detailed profiling of populations and benefiting from existing extensive data of internal migration in Peru. Over the 7-year study period we observed an increase in the prevalence of obesity in all study groups, with the intra-urban migrants and rural-to-urban migrants having the highest rates of obesity (around 21% in 2012); those remaining in rural had the lowest rates (10% in 2012). The largest increase in obesity rates over the period was observed among the intra-rural migrants, from 6.6% in 2005 to 13% in 2012. We found that, relative to rural stayers, all those who exposed to urban environments, including urban-to-rural migrants, had from two- to three-fold greater odds of obesity. These raised probabilities are also observed in the intra-rural migrant group, which had 42% higher obesity odds relative to rural non-migrants. Taken together, these findings indicate that both exposure to urban environments and migration are associated with higher odds of obesity, and our study advances the characterization of the association of migration-obesity profiles.

Given the rapid pace of urbanization in most LMICs, driven largely by rural to urban migration, these findings help illustrate the impact of urbanization, and the exposure to urban settings more precisely, is not uniform across groups; important changes are occurring across the spectrum of migration profiles: while migrants to an urban setting have much higher rates of obesity, the increase in the magnitude of obesity prevalence is considerably greater among intra-rural migrants in Peru. In general, any pattern of migration, i.e. migration into an urban or into a rural area, is associated with obesity.

We have previously shown that exposure to urban environments is linked to higher odds of obesity, with a clear relationship with duration of residence in urban settings. This supports comparable findings with other studies focused on rural-to-urban only migrants in Peru [2, 11], and in other LMICs undergoing internal migration [12]. In this study, we were able to also observe a relationship, albeit smaller, in the same direction of increased obesity with rural migration. This means that any migration, irrespective of the host environment, is associated with increased odds of obesity. Although this appears counter-intuitive, particularly for intra-rural migrants, as most of the literature signals that exposure to rural areas exert a protective factor for obesity. Our study adds further granularity into this human-environment exposure in that migration per se introduces changes, some of them behavioral ones, that override the protective factor of being exposed to a rural area. Hence, internal migration per se, and not only rural-to-urban migration, becomes a risk factor for obesity.

A recent population-based study in India, also using cross-sectional data, found higher obesity odds among intra-urban and rural-to-urban women migrants, though the results for men were not conclusive [13]. That said, in contrast to our results, Varadharajan et al. found that urban-to-rural migration in women was a protective factor against obesity [13]. These differences could be due to the fact that India is less urbanized than Peru [14] and significantly poorer [15], and thus migrants in Peru are more exposed to the unhealthy lifestyles associated with prolonged exposure to urban living. Our results, including prospective studies [2], could signal an alert for a developing obesity epidemiological scenario in India and other LMICs which while still less urbanized than Peru [14], are indeed undergoing urbanization.

Our results could be explained by differences in socioeconomic status amongst migration groups. It is often argued that migration towards urban sites is motivated and facilitated by better socio-economic status i.e. people migrate to look for work or better living conditions in urban areas, but also that those able to migrate are relatively better off [16, 17]. In fact, a previous cross-sectional study with Peruvian rural-to-urban migrants found that lower socio-economic status and the conditions of poverty were associated with lower odds of obesity [18]. In a way, this reflects the fact that obesogenic drivers closely follow lifestyles associated with, or made possible by, a higher socio-economic status. From a pragmatic point of view, our findings support calls to promote obesity-prevention strategies in rural areas and in all those sites undergoing economic improvement, to stave off potential obesity epidemics and the health problems and costs this entails [19].

Over the last decades, Peruvians have moved from rural to urban areas seeking of better opportunities: improved education, access to services and housing [20, 21]. However, this is not always possible and rural-to-urban migrants usually move to the outskirts of Lima (capital city) and build their own houses, lacking access to basic services (e.g., potable water). Overall, rural-to-urban migrants have been characterized as: young rural women, mostly indigenous, illiterate, with dearth of knowledge about living in the city, with poor household background, and mostly working in household and agriculture [22]. Therefore, what could have driven our results is probably the exposure to urbanization, either because of rural-to-urban migration or due to the increasing urbanization of rural areas, rather than the migrants having different characteristics than their predecessors.

Our study has found that different migration profiles are associated with greater or lesser odds of being and becoming obese. Increases in obesity rates at the population level reflect shifting BMI levels, where increases of up to 5 Kg/m^2^ units have been linked to a three-time greater risk of developing diabetes [23]. Hence, if we were to apply these assumptions to a LMIC like Peru, our analysis would support both the understanding *and* forecasting of the epidemic of diabetes, a costly cardio-metabolic risk factor associated to loss of productivity and early mortality [24]. Understanding both urban and rural settings merit attention and is important. Whilst greater focus has been placed on public health and nutrition in urban settings, no doubt due to the greater perceived burden, a significant proportion of the population are still based in rural areas; our findings could well inform preventive strategies and health messages which could be applied to tackle growing obesity in both rural and urban settings. Also important is developing public health messages to parents and grandparents to stave off obesity in young children, given the rising prevalence of obesity across the world [19].

Our study benefitted from a large sample size from a population-based survey of Peruvian women, with almost annual data from 2005 to 2012 and with a dynamic within-country migration profile. The use of the Peru DHS is an asset because it allows for an examination of the geographic and sociodemographic differences in health outcomes according to internal migration profiles. We acknowledge the limitations of this study. We were unable to control the regression models for the initial conditions of the population, particularly participants’ BMI *prior* to migration. However, our aim was not to assess the longitudinal trajectory of individual BMI or to assess changes over time for a given group. We aimed, instead, to assess the effect of internal migration and to see if such an effect varied over a period of time. Also, our regression models did not account for other overweight determinants, such as diet or physical activity, because these variables were not available. Future studies on trajectories of weight should include these variables, particularly when assessing people changing from rural to urban sites, or vice versa. For some migration categories, i.e., urban-to-rural or intra-rural migrants, there were small numbers in the sample (Table 1), and this could have compromised the statistical power of the regression analysis. In addition, other co-variables also had small sample size in some categories (e.g., ethnicity). Thus, results for these small groups deserve a cautious interpretation. Nonetheless, this should not be regarded as a limitation, because our results are robust for rural-to-urban migration, which happens to be the most frequent migration. Finally, the large sample size available only included adult women aged 15–49 years in the study, given there were no similar data on men for the same time period. Therefore, our results partially inform of the overall scenario in Peru, without including men or older women.

## Conclusion

In conclusion, urban exposure is associated with higher probability of being obese. Although rural exposure seemed to be a protector factor, the effect among urban-to-rural migrants diminished with duration in place of residence. The increasing odds of obesity across all groups in Peru forecast an alarming scenario given a high BMI is a risk factor for cardio-metabolic conditions. The fact that the rate of increase of obesity is highest for rural-to-urban migrants should be a concern for public health, given rural areas constitutes the main reservoir of potential migrants across LMICs. Obesity prevention strategies could take some lessons from rural areas, as these settings seem to protect against high BMI, but given that obesity prevalence rates in rural areas are also rising rapidly suggests a need to monitor the nutrition and demographic dynamics in both urban and rural settings.

## Abbreviations

95%CI: 95% Confidence Intervals
BMI: Body Mass Index
DHS: Demographic and health survey
LMIC: Low- and middle-income countries
